# Folk-Psychological Interpretation of Human vs. Humanoid Robot Behavior: Exploring the Intentional Stance toward Robots

**DOI:** 10.3389/fpsyg.2017.01962

**Published:** 2017-11-14

**Authors:** Sam Thellman, Annika Silvervarg, Tom Ziemke

**Affiliations:** ^1^Cognition & Interaction Lab, Department of Computer and Information Science, Linköping University, Linköping, Sweden; ^2^Interaction Lab, School of Informatics, University of Skövde, Skövde, Sweden

**Keywords:** human–robot interaction, folk psychology, social interaction, intentional stance, attribution theory, intentionality ascription, behavior explanation, social robots

## Abstract

People rely on shared folk-psychological theories when judging behavior. These theories guide people’s social interactions and therefore need to be taken into consideration in the design of robots and other autonomous systems expected to interact socially with people. It is, however, not yet clear to what degree the mechanisms that underlie people’s judgments of robot behavior overlap or differ from the case of human or animal behavior. To explore this issue, participants (*N* = 90) were exposed to images and verbal descriptions of eight different behaviors exhibited either by a person or a humanoid robot. Participants were asked to rate the intentionality, controllability and desirability of the behaviors, and to judge the plausibility of seven different types of explanations derived from a recently proposed psychological model of lay causal explanation of human behavior. Results indicate: substantially *similar judgments* of human and robot behavior, both in terms of (1a) ascriptions of intentionality/controllability/desirability and in terms of (1b) plausibility judgments of behavior explanations; (2a) *high level of agreement* in judgments of robot behavior – (2b) slightly lower but still largely similar to agreement over human behaviors; (3) *systematic differences* in judgments concerning the plausibility of goals and dispositions as explanations of human vs. humanoid behavior. Taken together, these results suggest that people’s intentional stance toward the robot was in this case very similar to their stance toward the human.

## Introduction

People’s understanding of social interactions is based on, or at least influenced by, folk-psychological interpretations of observed behavior (e.g., Anscombe, 1957; Heider, 1958; Davidson, 1963; Goldman, 1970; Dennett, 1971; Buss, 1978; Searle, 1983; Audi, 1993; Malle, 2004). This applies to interactions with other people, to interactions with many types of animals, and presumably also to interactions with artificial agents, such as social robots, virtual agents, or automated vehicles (e.g., Waytz et al., 2014). In human-robot interaction (HRI) research, for example, there has been a substantial interest in the role of intentions in recent years (e.g., Wykowska et al., 2015, 2016; Admoni and Srinivasa, 2016; Vernon et al., 2016). So far, however, there has been very little comparative research on how people actually interpret the behavior of different types of artificial agents, and how this compares to human–human social interaction. Wiese et al. (2012), for example, showed that people were more inclined to engage in joint attention with a robot when treating it as an intentional system, i.e., a system interpreted as having intentions, goals, and similar mental states. Sciutti et al. (2014) also showed that people shift their gaze toward perceived “goals” of robot actions prior to the execution of the actions themselves, which suggests that people view robot behavior as goal-directed. Furthermore, goal-directed actions, such as grasping a wine glass by the stem or placing a lid on a salt jar, are known to evoke similar mirror system activity in humans when exhibited by robots as when performed by humans (e.g., Gazzola et al., 2007; Oberman et al., 2007). This indicates that people’s interpretations of robots and humans as goal-directed agents are supported by the same or overlapping biological mechanisms. Chaminade et al. (2012), on the other hand, argue that “the neural correlates of taking the intentional stance” are not activated in interactions with artificial agents. Their experiments, however, were limited to a relatively simple rock-paper-scissors scenario. Hence, overall, relatively little is known regarding to what degree the underlying psychological and biological mechanisms overlap or differ for such a broad variety of different types of natural and artificial agents. The research reported here, investigating people’s folk psychological explanations of human vs. humanoid behavior, is intended to make a small contribution toward closing that gap.

As also mentioned in the call for papers for the “*Intentions in HRI”* research topic (which this paper is submitted to), different parts of the literature in the cognitive sciences provide us with at least two possible working hypotheses. On the one hand, ever since Heider and Simmel’s (1944) seminal psychological research on attribution, it is well known that people tend to interpret the movement of even very simple geometric shapes in terms of more or less human-like actions and intentions. This could be taken to point to the existence of universal schemata and mechanisms that are applied to any type of system that can be interpreted as an intentional ‘agent’, relatively independent of what that agent might look like (in Heider and Simmel’s case these were simple circles, triangles, etc.). On the other hand, much social neuroscience research in the last two decades, in particular the discovery of the mirror (neuron) system (e.g., Rizzolatti and Craighero, 2004; Thill et al., 2013), seems to indicate that similarities and differences in embodiment/morphology might play a crucial role in the understanding of others’ actions and intentions. Buccino et al. (2004), for example, ran experiments on people’s perception of mouth actions carried out by other people, monkeys and dogs, and their results indicated that (1) the same brain areas were activated by the recognition of both conspecifics- and non-conspecifics’ actions, but (2) there was a gradual decrease in activation as the species gets more morphologically distant from the human observer (i.e., less activity for monkey than for human actions, and least activity for dog actions). This could be taken to indicate that humans might be able to understand the behavior of human-like robots more easily than, for example, the behavior of autonomous lawnmowers or automated vehicles.

The notions of ‘intention’ and ‘intentionality’ arguably play a central role in how we interpret the behavior of other agents – natural and artificial. Malle et al. (2001, p. 1) note that the concept of intentionality “brings order to the perception of behavior in that it allows the perceiver to detect structure – intentions and actions – in humans’ complex stream of movement … [and] supports coordinated social interaction by helping people explain their own and others’ behavior in terms of its underlying mental causes”. In the context of human-robot interaction (HRI), it might be worth noting that the term ‘intentionality’ is used in at least two overlapping, but different senses. Searle (1999, p. 85), for example, characterizes the intentionality of an *individual* agent’s own mental states as follows:

“The primary evolutionary role of the mind is to relate us in certain ways to the environment, and especially to other people. My subjective states relate me to the rest of the world, and the general name of that relationship is “intentionality.” These subjective states include beliefs and desires, intentions and perceptions, …. “Intentionality,” to repeat, is the general term for all the various forms by which the mind can be directed at, or be about, or of, objects and states of affairs in the world.”

However, Searle (1999) also stresses that competence in predicting and explaining (human) behavior involves being able to both recognize *others* as *intentional* beings, and interpret others’ minds as having “intentional states,” such as beliefs and desires. This is what Dennett (Dennett, 1989, p. 17) refers to as the *intentional stance*, i.e., the ascription of intentions and intentional states to other agents in a social context. His explanation illustrates the role of folk-psychological reasoning in interpreting the behavior of others:

“Here is how it works: first you decide to treat the object whose behavior is to be predicted as a rational agent; then you figure out what beliefs that agent ought to have, given its place in the world and its purpose. Then you figure out what desires it ought to have, on the same considerations, and finally you predict that this rational agent will act to further its goals in the light of its beliefs. A little practical reasoning from the chosen set of beliefs and desires will in most instances yield a decision about what the agent ought to do; that is what you predict the agent will do.”

As the example of Heider and Simmel’s (1944) work nicely illustrates, it might be worth noting that taking the intentional stance toward some object – or ‘agent’ – in Dennett’s sense is not necessarily the same as believing that that ‘agent’ actually has genuine intentionality in Searle’s sense. This is relatively obvious for cartoon characters (cf. Ziemke and Thill, 2014): when, for example you watch a Donald Duck movie in which Donald is angrily chasing the chipmunks Chip and Dale, who are trying to steal his pancakes, you of course understand the mental states, intentions, etc. that are implied by the movie, without necessarily believing that Donald Duck really exists, has agency, loves pancakes, and is angry with the Chip and Dale. This is important to keep in mind in the human–robot interaction (HRI) context, where a human observer’s *folk-psychological* interpretation of a robot’s behavior in some social context needs to be understood independent from the scientific, technological or philosophical considerations underlying the construction of that robot.

The general long-term motivation behind our research is to further our understanding of how, when and why people take the intentional stance – in Dennett’s broad sense – toward robots and other types of autonomous systems, and what the underlying folk-psychological mechanisms might be. The present paper, or any *one* paper, can of course not answer this broader research question and resolve the many issues involved. Instead, we here focus on some initial experiments comparing how people interpret human and humanoid behavior, and to what degree they make use of shared folk-psychological mechanisms in this. Hence, the more specific research questions we focus on in this paper are the following:

• Q1: How similar or different are people’s judgments of the intentionality, controllability and desirability of human vs. humanoid behavior?
• Q2: How similar or different are people’s judgments of the causes of the behavior of humans vs. humanoids, in terms of the underlying folk-psychological mechanisms, and how these overlap or vary for different behavior types?
• Q3: How much do people agree or disagree in their judgments of humanoid robot behavior compared to judgments of human behavior?

As discussed in more detail in section “Background,” the experiments reported here draw on social psychological literature on attribution, including empirically validated models such as the recently proposed model of people’s lay causal explanations of human behavior published by Böhm and Pfister (2015) in this journal. Section “Method” then describes in detail the methods and materials used. Results are analyzed in section “Results,” and discussion and conclusions are presented in section “Conclusion and Discussion.”

## Background

Social interaction hinges crucially on people’s ability to infer the reasons behind others’ actions: false inferences lead to misguided explanations and predictions with failed coordination and conflict as potential outcomes. In particular, attribution theorists have stressed the fact that people’s judgments of behavior as intentional or unintentional are particularly consequential to how they experience social interactions, and often determine whether they are viewed as positive or negative (e.g., Heider, 1958; Malle, 2004). For example, the outcome of accidentally bumping into someone in the street may be rather different depending on whether the person bumped into recognizes the behavior as intentional or unintentional. Fortunately, humans are adept intention detectors: we show substantial agreement in judgment when asked to differentiate among intentional and unintentional behaviors (Malle and Knobe, 1997), we make accurate judgments of intentional behavior from observing motion cues alone (Barrett et al., 2005), and insights from developmental psychology suggest that children are able to detect intentions by age one (Astington, 2001). It is important to note, however, that people’s expertise in judging human behavior does not necessarily generalize to the case of robots. This is a potential issue for human-robot interaction (HRI) research which strives toward designing robots that are able to interact with humans in daily life (e.g., Fong et al., 2003; Li et al., 2011).

There are now numerous studies that show that people treat robots *as if* they were living creatures endowed with mental states, such as intentions, beliefs, desires, and cite reasons as opposed to causes as explanations for their actions (e.g., Duffy, 2003; Krach et al., 2008; Waytz et al., 2010; Özdem et al., 2017). Treating robots as intentional systems also seems to benefit human–robot interactions. For instance, Wiese et al. (2012) showed that people were more willing to engage in joint attention with a robot when they adopted the intentional stance toward it. Furthermore, in some cases people might *have to* adopt the intentional stance toward robots. Philosopher Daniel Dennett proposed that people use three different strategies to understand and predict behavior. Dennett (1971, pp. 87–89) lays out an explanation of the three “stances” using the example of trying to predict the moves of a chess-playing computer. Taking the *physical stance* means making predictions based on the physical state of the particular object and the knowledge we have of the laws of nature, such as when predicting that the snow-covered roof of a building is about to collapse. According to Dennett “one seldom adopts the physical stance in dealing with a computer just because the number of critical variables in the physical constitution of a computer would overwhelm the most prodigious calculator.” In adopting the *design stance*, people rely on their knowledge of the design of the object of prediction: “if one knows exactly how the computer is designed (including the impermanent part of its design: its program) one can predict its designed response to any one move one makes by following the computation instructions of the program.” Dennett notes that complex systems, such as the best chess-playing computers, are “practically inaccessible to prediction from either the design stance or the physical stance; they have become too complex for even their own designers to view from the design stance.” Instead, Dennett contends that “a man’s best hope of defeating such a machine in a chess match is to predict its responses by figuring out as best as he can what the best or most rational move would be, given the rules and goals of chess.” This is what Dennett calls taking the *intentional stance*, i.e., to explain and predict the behavior of a system by relying on ascribing beliefs, desires, intentions, and other “Intentional idioms” (cf. Dennett, 1971, p. 87) to it, and by assuming that it will act rationally in accordance with those beliefs and desires. Complex robotic systems are expected to handle a myriad of problems arising in day-to-day encounters (e.g., traffic scenarios), some of which also require social interaction with people. Arguably, the behavior of such systems will in many cases be difficult, if not impossible, to predict and explain from a design standpoint. Hence, people will in many cases have nothing to rely on in their interaction with such systems beyond their interpretations of them as intentional systems. It is important therefore, that the intentional stance toward robots is studied in detail. This involves not only questions of *why* and *when* people take the intentional stance toward robots, but also how people interpret different types of autonomous systems (e.g., humanoid robots, autonomous vehicles, drones etc.) *qua* intentional systems and how this compares to the human case.

**FIGURE 1:**
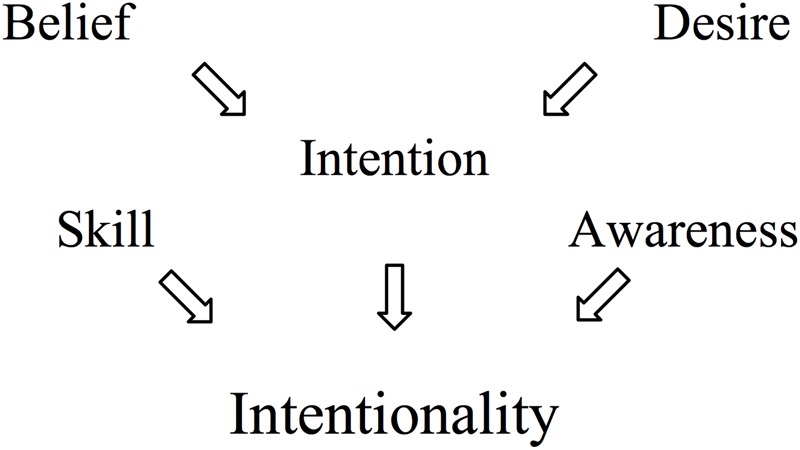
The constituents of the folk concept of intentionality. Adapted from Malle and Knobe (1997).

As far as the human case goes, there is plenty of literature on the ascription of mental states to humans within the social psychological literature on attribution (e.g., Heider, 1958; Jones and Davis, 1965; Kelley, 1967). However, only a few of these theories are based on empirical investigations of people’s actual ascriptions to others. One such model, called *the folk concept of intentionality* (Malle and Knobe, 1997), specifies the constituent components of intentional behavior (see **Figure 1**). The authors built this model from asking people to rate different behaviors (as described verbally on paper) along various dimensions, such as whether they are intentional or not, and through acquiring definitions of folk-psychological concepts from people’s answers to free-response questions. The folk concept of intentionality thus encapsulates the preconditions for when people view human behavior as intentional (as indicated by the arrows in **Figure 1**). People recognize behavior as intentional only when they see: a *desire* for an outcome; *beliefs* about an action that leads to that outcome; an *intention* to perform the action; *skill* to perform the action; and *awareness* of fulfilling the intention while performing the action, i.e., “knowing what one is doing while doing it” (Malle and Knobe, 1997, p. 108).

**FIGURE 2:**
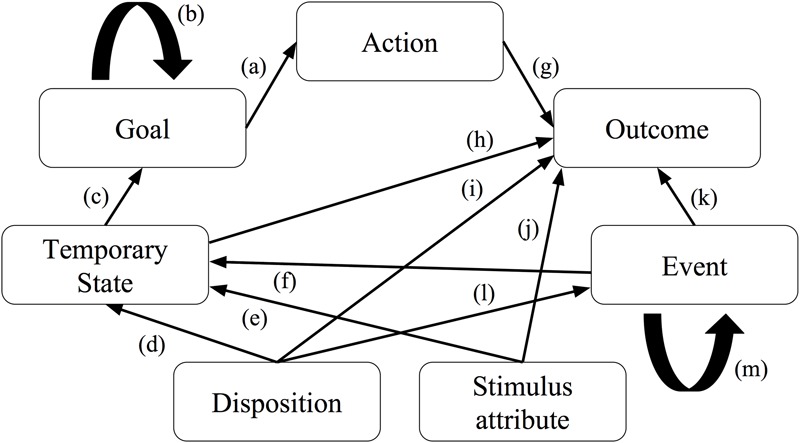
Behavior and explanation types (boxes) and inference rules (arrows) in the Causal Explanation Network (CEN) model. Adapted from Böhm and Pfister (2015).

Empirical attribution theorists have also been concerned with how people explain the behavior of others. Based on previous attribution research, Böhm and Pfister (2015) proposed the *causal explanation network* (CEN) model (cf. **Figure 2**), a model of people’s folk-psychological causal explanations of human behavior. The model consists of seven cognitive categories used for both behavior encoding and explanation: *goals, intentional actions, action outcomes, temporary states, dispositions, uncontrollable events*, and *stimulus attributes*. The relations between the different categories, expressed as arrows in the model, are assumed to reflect the causal explanations of the various behaviors. For instance, rule (a) in the model states that actions are explained with reference to goals, and rule (b) states that a goal can be explained by a higher order goal. Böhm and Pfister performed a series of empirical experiments to validate the CEN model and found that people’s explanations of various behaviors where in line with the categories of the model. Furthermore, behaviors and explanation types that are related in the model were used more often and with shorter reaction times. Hence, they concluded that “the seven categories postulated in the CEN model seem to reflect the cognitive concepts that make up the lay theory of behavior” (ibid., p. 12).

For similar reasons as discussed above, it is unclear whether people see the seven cognitive categories featured in the CEN model as plausible causes of robot behavior. More generally, the proposition held by many attribution theorists that people’s social interactions are guided by shared folk-psychological mechanisms (such as captured by the aforementioned models) is not necessarily only a matter of “either people do or they don’t” – as argued previously, these mechanisms might differ for different types of agents. We believe that this topic represents largely unchartered terrain, and upon searching previous literature we found little existing methodology to adapt in our research design. We therefore here explore a new method.

## Method

This study was carried out in accordance with institutional guidelines, with written informed consent from all participants in accordance with the Declaration of Helsinki.

### Participants and Design

Ninety-three university students with different curricula including economics, computer science, cognitive science, and history, were asked to individually complete a social psychological survey concerning how people interpret and explain behaviors. The only precondition for participation was (self-assessed) proficiency in the Swedish language. In a between-subjects study design, one group of participants were presented with human behavior stimuli and another group of participants were presented with humanoid behavior stimuli.

Data from three participants were excluded from the study for the following reasons: one person reported not having paid attention to the robot behavior image and consequently rated the behaviors with a human actor in mind, a second person reported having underestimated his or her proficiency in Swedish when being asked prior to the study, and a third person only filled out one page of the questionnaire without giving any reason as to why. The exclusion of three participants from the study resulted in a sample size of 47 participants in the human behavior condition and 43 participants in the humanoid behavior condition, equaling a total of 90 participants [*M*_(age)_ = 24, *SD* = 3.3 years., 52% women, 47% men, 1% unspecified].

### Stimuli

Perhaps the most widely used method in empirical attribution research is to expose people to written descriptions of particular behavior (e.g., “John helped the old lady cross the street”) and to ask them to rate the behavior on some parameter using a questionnaire. We wanted to employ this method in studying humanoid-enacted behavior. However, if participants were provided with written behavior descriptions only it would have been possible (if not likely, given that people have no experience interacting with robots) that some of the participants would think of a person, not a humanoid actor, when judging the behaviors. To avoid this risk, we decided to complement written behavior descriptions with concrete images of a humanoid robot enacting the described behaviors.

The setting of the behaviors had to be plausibly naturalistic. Also, individual behaviors had to be possible to enact in ways that would render them believable in static pictures. With these considerations in mind, we based our selection of stimuli on a kitchen scenario. We decided to use the CEN model (Böhm and Pfister, 2015) as a starting point and adopted the model’s four specified behavior types as a basis for our stimuli: *actions, outcomes, events*, and *temporary states*. It is important to note that this decision was motivated by the need to generate a reasonably diverse set of behavior stimuli and that we were not, for the purpose of the present study, concerned with assessing the validity of the model. To account for positivity bias, i.e., the tendency to make different attributions for positive and negative events (e.g., Mezulis et al., 2004), each of the four behavior types were created in a desirable and an undesirable version.

**FIGURE 3:**
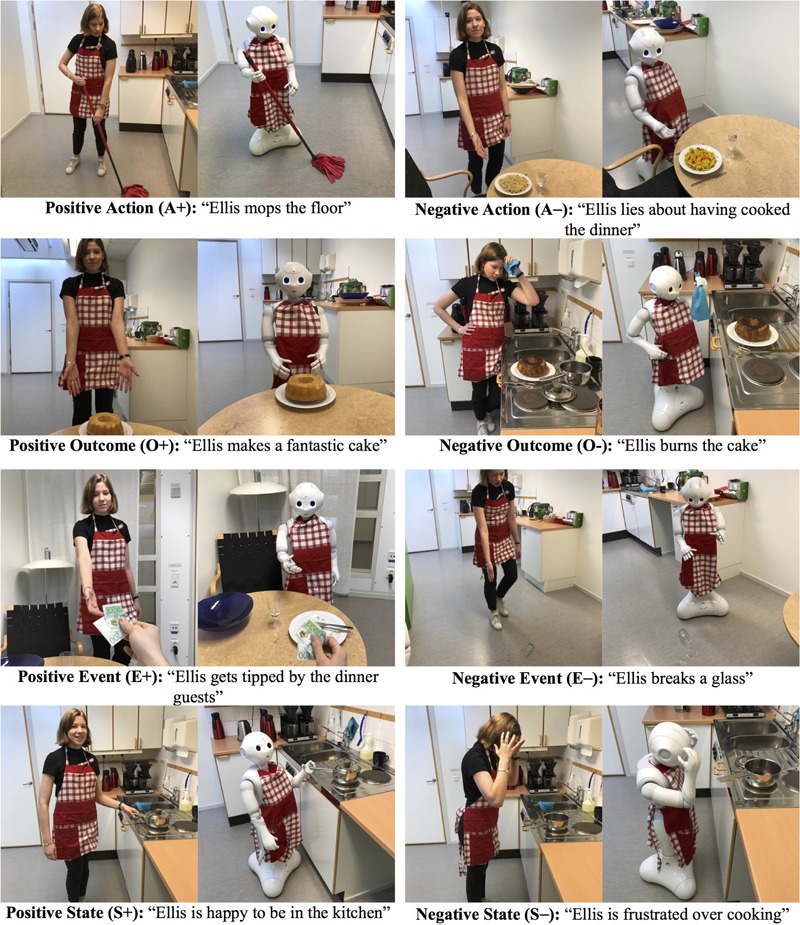
Behavior descriptions and images used as experimental stimuli. The person in the images, Sofia Thunberg, has given written consent for publication.

The above criteria resulted in eight individual stimuli per experimental condition (human and robot) as shown in **Figure 3**:

• Positive action (A+): “Ellis mops the floor”
• Negative action (A-): “Ellis lies about having cooked the dinner”
• Positive outcome (O+): “Ellis makes a fantastic cake”
• Negative outcome (O-): “Ellis burns the cake”
• Positive event (E+): “Ellis gets tipped by the dinner guests”
• Negative event (E-): “Ellis breaks a glass”
• Positive event (S+): “Ellis is happy to be in the kitchen”
• Negative state (S-): “Ellis is frustrated over cooking”.

### Measures

Each participant was provided with a questionnaire containing eight individual pages. Each page contained, from top to bottom: one of the eight behavior images pertaining to the experimental condition (human or robot) covering approximately one third of the page width, the verbal description of the behavior, and ten Likert-style questions with the option to select one of seven ordinal values ranging from “not at all” to “completely.” The first three questions concerned participants’ interpretations of the behaviors as intentional, controllable, and desirable, and were given in Swedish^1^ in the form *“Rate to what extent Ellis’ behavior is X”* where X was *intentional, under Ellis’ control*, and *desirable*, respectively. The following seven questions concerned judgments of the plausibility of various causal explanations being true of the behavior they explain. Again, for the purpose of generating a reasonably diverse set of causal explanations we based our selection on the explanations types specified in the CEN model (Böhm and Pfister, 2015). The seven questions were translated into Swedish and given in the form *“Rate how plausible it is that the cause of Ellis’ behavior is X”*, where X was a *conscious goal*, an *action*, an *outcome*, an *uncontrollable event*, a *temporary state* (psychological or physical), a *disposition*, and an *attribute* of someone or something in Ellis’ environment. The eight questionnaire pages were presented to participants in pseudo-randomized order to balance the influence of potential confounds related to prolonged exposure to similar stimuli and task (repeatedly filling out similar questions) or transfer of earlier judgments to subsequent judgments. Although confounding effects are impossible to wholly eliminate, this measure was taken to ensure that the potential influence of such effects would be similar for both conditions and all ratings included in the questionnaire.

### Procedure

Prior to participation the experimenter showed each participant the consent form, a page containing demographical questions, and the first page of the questionnaire containing experimental stimulus. The experimenter explained that the questions on each page concerned the properties and plausible causes of the behaviors as they were described and enacted by the actor depicted on the form, and that the task of the participant was to answer the questions according to their best ability. The demographical questions concerned age, gender and self-assessed technical competence (rated on a seven-value Likert sequence from “Low” to “High”). After the above instructions were given, all participants gave informed consent and proceeded to fill in the questionnaire. Upon completion, participants were made aware of the second experimental condition and the purpose to compare people’s judgments of human and robot behavior and were given an opportunity to ask questions and comment on the study.

## Results

The results of the study are grouped into three sections based on the above stated research questions: section “Behavior Interpretations” reports on the analysis of participant ratings of the intentionality, controllability and desirability of behaviors; section “Plausibility Judgments of Behavior Explanations” concerns plausibility-ratings of explanations for behaviors; and section “Agreement in Ratings of Human vs. Humanoid Behavior” reports on the level of agreement in participants’ judgments. The two experimental conditions (human vs. humanoid actor) did not significantly differ with regard to age or gender, as assessed by Independent-Samples *T*-Test and Fisher’s Exact Test, respectively. However, participants’ self-assessed technical competence was higher in the human condition (*M* = 4.80, *SD* = 1.21) than the humanoid condition (*M* = 4.20, *SD* = 1.23), *U* = 659, *z* = -2.209, *p* < 0.05. We also include in the **Appendix** a matrix of correlations among aggregated ratings of the ten questionnaire items across all eight behaviors in each experimental condition.

Likert methodology is one of the most commonly used methodologies in all fields of research, however, there is still some debate over the issue of how to treat Likert-style (ordinal) data in inferential statistics (see Jamieson, 2004; Carifio and Perla, 2008; Norman, 2010). For this study, which utilizes 7-degree Likert-style questions (no scale) with bipolar labels only, we chose the mean as the measure of central tendency in participant ratings and standard deviation to express variability. Group comparisons were conducted using parametric null hypothesis significance testing following the recommendations in Norman (2010). Bar charts are presented with 95% CI error bars and asterisks (^∗^) indicating statistically significant differences at *p* < 0.05 throughout the result section.

### Behavior Interpretations

#### Intention

**FIGURE 4:**
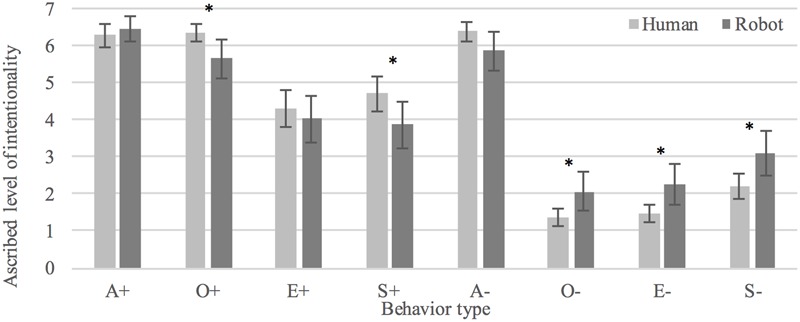
Interpretations of human and robot behaviors as intentional. ^∗^Denotes a statistically significant difference (*p* < 0.05).

There was no statistically significant overall difference in participants’ interpretations of the behaviors as *intentional* between human (*M* = 4.14, *SD* = 2.37) and humanoid (*M* = 4.16, *SD* = 2.41) conditions, *t*(709.323) = -0.119, *p* = 0.905. Positive behaviors (+) were in general seen as more intentional when enacted by the human (*M* = 5.40, *SD* = 1.66) as compared to the humanoid (*M* = 5.00, *SD* = 2.12), *t*(323.722) = 1.999, *p* < 0.05, *d* = 0.22. Ratings of negative behaviors (-) did not significantly differ between conditions, *t*(351.272) = 1.835, *p* = 0.067. However, three out of four negative behaviors (O-, E-, and S-) were rated as less intentional in the humanoid condition as compared to the human condition (*p* < 0.05) with effect sizes of *d* = 0.63, 0.65, and 0.59, respectively. Participants interpreted positive behaviors as significantly more intentional than negative behaviors in both human, *t*(186) = 14.804, *p* < 0.0005, *d* = 1.08, and humanoid conditions, *t*(171) = 8.075, *p* < 0.0005, *d* = 0.65. See **Figure 4** for an overview of judged intentionality of the eight behaviors in human and humanoid conditions.

#### Control

**FIGURE 5:**
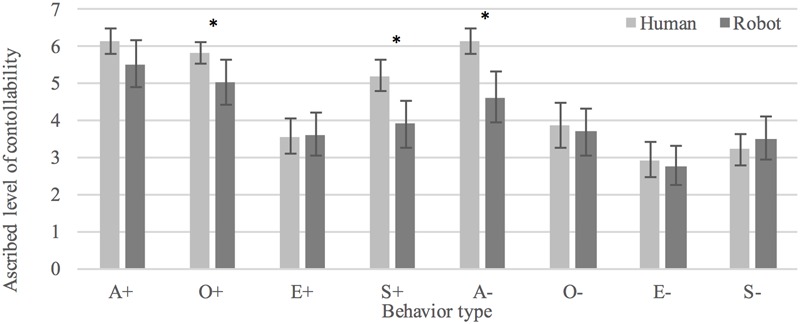
Interpretations of human and robot behaviors as controllable. ^∗^Denotes a statistically significant difference (*p* < 0.05).

Overall, participants viewed behavior as more *controllable* when enacted by the human (*M* = 4.61, *SD* = 1.97) as compared to the robot (*M* = 4.08, *SD* = 2.20), *t*(690.319) = 3.382, *p* < 0.005, *d* = 0.26. Positive behaviors were seen as more controllable than negative behaviors in the human condition, *t*(319.414) = 3.185, *p* < 0.005, *d* = 0.36. There was no statistically significant difference between conditions in rated controllability of negative behaviors, *t*(357) = 1.768, *p* = 0.078. Participants interpreted positive behaviors as significantly more controllable than negative behaviors in both human, *t*(186) = 7.299, *p* < 0.0005, *d* = 0.53, and humanoid conditions, *t*(171) = 4.572, *p* < 0.0005, *d* = 0.35. See **Figure 5** for an overview of judged controllability of the eight behaviors in human and humanoid conditions.

#### Desirability

**FIGURE 6:**
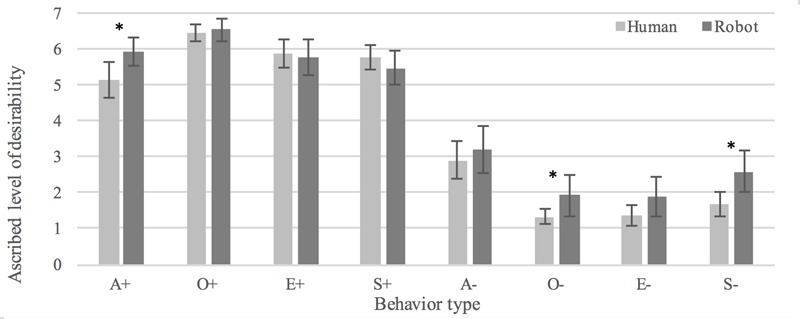
Interpretations of human and robot behaviors as desirable. ^∗^Denotes a statistically significant difference (*p* < 0.05).

There was no statistically significant overall difference between participants’ interpretations of the behaviors as *desirable* in human (*M* = 3.82, *SD* = 2.42) vs. humanoid (*M* = 4.17, *SD* = 2.51) conditions, *t*(717) = -1.871, *p* = 0.062. Judgments of positive behaviors did not significantly differ between conditions, *t*(358) = -0.770, *p* = 0.422. However, negative behaviors were in general seen as less desirable when enacted by the human (*M* = 1.82, *SD* = 1.37) as compared to the robot (*M* = 2.41, *SD* = 2.05), *t*(294.542) = -3.141, *p* < 0.005, *d* = 0.37. Participants rated positive behavior as more desirable than negative behavior in both the human, *t*(186) = 24.922, *p* < 0.0005, *d* = 1.82, and the humanoid condition, *t*(171) = 2.195, *p* < 0.0005, *d* = 1.42, which validates the stimuli as examples of positive and negative behaviors. See **Figure 6** for an overview of judged desirability of the eight behaviors in human and humanoid conditions.

### Plausibility Judgments of Behavior Explanations

Participants were asked to rate the extent to which the behaviors enacted by the two types of agents were plausibly explained by seven different types of causes. The ratings were similar between human and humanoid conditions in 46 out of 58 individual cases as assessed using multiple independent samples *t*-tests (**Table 1**). The twelve statistically significant differential effects were all *moderately* sized, ranging from *d* = 0.48 to *d* = 0.68 (Cohen, 1988). While we will not discuss individual effects in detail in this paper, we note here that nine out of the twelve differential effects pertained to ratings of the plausibility of *goal* and *disposition* as explanations of behavior (we will return to this finding in the discussions section).

**Table 1:** Mean ratings of the plausibility of explanation types (rows) for human (left value in cell) and humanoid (right value in cell) behaviors (columns).

	A+	O+	E+	S+	A-	O-	E-	S-
Goal	5.7/6.0	6.4/5.8	5.3/5.0	4.8/4.8	5.8/5.4	1.4/2.3	1.6/2.5	2.2/3..82
Action	5.8/6.0	6.0/5.8	4.8/5.0	5.0/4.6	5.5/5.5	4.1/4.5	4.6/4.7	4.4/4.1
Outcome	5.4/5.7	5.9/5.9	6.0/5.7	5.1/5.2	5.6/5.5	5.7/5.8	5.4/5.1	5.8/5.7
Event	2.7/2.3	2.3/2.2	3.2/3.4	2.8/3.0	2.3/2.8	4.1/3.5	4.6/3.9	4.8/3.7
Temp. state	2.7/2.8	3.0/3.2	2.9/3.1	4.6/4.4	4.0/3.8	2.9/3.9	3.6/3.6	5.1/4.6
Disposition	3.7/3.4	4.3/3.3	4.1/3.9	5.0/4.1	5.1/4.8	3.4/2.5	3.6/2.6	4.4/3.5
Stimulus attr.	3.7/4.7	3.2/3.8	5.0/5.0	3.9/4.4	3.8/4.3	3.3/3.7	3.3/3.5	3.8/4.3

*Statistically significant differences between groups (p < 0.05) are marked in inverted colors. The behavior types were: action (A), outcome (O), event (E), and temporary state (S) with positive (+) and negative (-) variations*.

### Agreement in Ratings of Human vs. Humanoid Behavior

Intraclass correlation coefficient (ICC) was used as the measure of agreement^2^ in participant ratings within each experimental condition. All ICC indices reported here are two-way random average measures, ICC (2, *k*), with an “absolute agreement” definition (Shrout and Fleiss, 1979). Lower and higher 95% confidence interval bounds are reported in brackets. We first report ICCs for interpretations of behavior (questions 1–3 in the form) followed by plausibility judgments of behavior explanations (questions 4–10). Guidelines for evaluating levels of intraclass correlation state that values below 0.40 can be considered as of *low* clinical significance, between 0.40 and 0.59 as *fair*, between 0.60 and 0.74 as *good*, and between 0.75 and 1.00 as *excellent* (Cicchetti, 1994).

**FIGURE 7:**
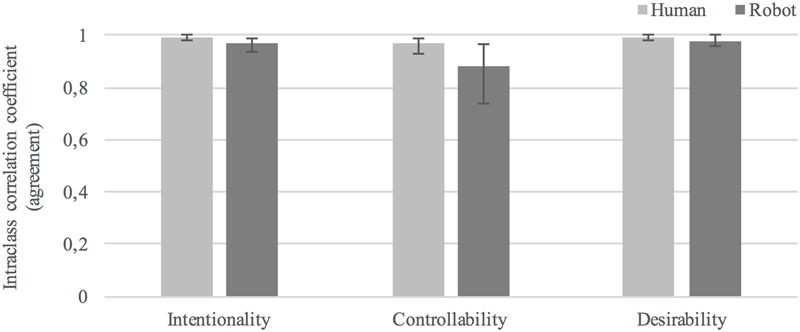
Average level of agreement in interpretations of human and humanoid behavior.

The average level of agreement in interpretations of behavior as: *intentional* was.99[0.98, 1.0], *F*(6,276) = 162.370, *p* < 0.0005, in the human condition and.97[0.94, 0.99], *F*(7,294) = 41.694, *p* < 0.0005, in the humanoid condition; *controllable* was 0.97[0.93, 0.99], *F*(6,276) = 39.309, *p* < 0.0005, in the human condition and 0.88[0.74, 0.97], *F*(7,294) = 11.336, *p* < 0.0005, in the humanoid condition; *desirable* was 0.99[0.98, 1.0], *F*(6,276) = 141.351, *p* < 0.0005, in the human condition and 0.98[0.96, 1.0], *F*(7,294) = 65.061, *p* < 0.0005, in the humanoid condition. These values all fall in the 0.75 to 1.00 range considered as “excellent” agreement according to guidelines for evaluation in Cicchetti (1994). Agreement was lower in judgments of humanoid than human behavior in three out of three cases (see **Figure 7**).

**FIGURE 8:**
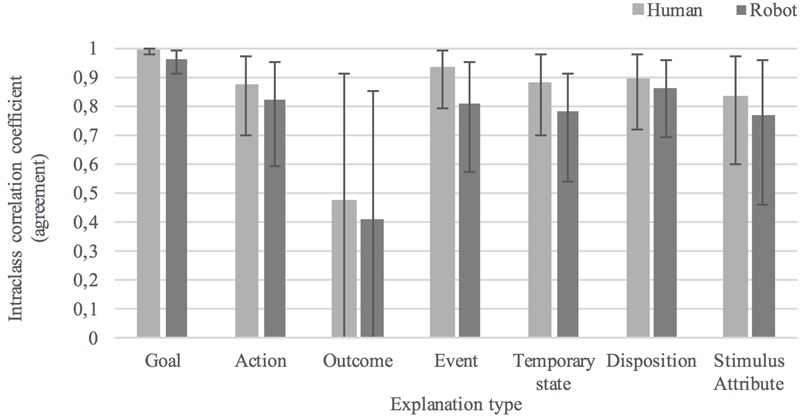
Average level of agreement in plausibility judgments of causes of human and robot behavior.

The average level of agreement in judgments concerning the plausibility that the cause of behavior was a(*n*): *goal* was 0.99[0.98, 1.0], *F*(6,276) = 117.764, *p* < 0.0005, in the human condition and 0.96[0.91, 0.99], *F*(7,294) = 32.930, *p* < 0.0005, in the humanoid condition; *action* was 0.87[0.70, 0.97], *F*(6,276) = 8.931, *p* < 0.0005, in the human condition and 0.82[0.59, 0.95], *F*(7,294) = 32.930, *p* < 0.0005, in the humanoid condition; *outcome* was 0.47[-0.18, 0.91], *F*(5,230) = 2.116, *p* = 0.064, in the human condition and 0.41[-0.21, 0.85], *F*(7,294) = 32.930, *p* = 0.081, in the humanoid condition; *event* was 0.93[0.79, 0.99], *F*(4,184) = 14.763, *p* < 0.0005, in the human condition and 0.81[0.57, 0.95], *F*(7,294) = 6.228, *p* < 0.0005, in the humanoid condition; *temporary state* was 0.88[0.70, 0.98], *F*(5,230) = 10.849, *p* < 0.0005, in the human condition and 0.78[0.54, 0.95], *F*(7,294) = 6.430, *p* < 0.0005, in the humanoid condition; *disposition* was 0.89[0.72, 0.98], *F*(5,230) = 10.871, *p* < 0.0005, in the human condition and 0.86[0.69, 0.96], *F*(7,294) = 10.429, *p* < 0.0005, in the humanoid condition; *stimulus attribute* of was 0.83[0.60, 0.97], *F*(5,230) = 7.921, *p* < 0.0005, in the human condition and 0.77[0.46, 0.96], *F*(5,210) = 5.796, *p* < 0.0005, in the humanoid condition. These results indicate excellent agreement in plausibility judgments for all types of causal explanations in both experimental conditions, with the exception of *outcome* for which no statistically significant effect was found. Agreement was higher in judgments of human than humanoid behavior for seven out of seven causal explanations. The between-group differences in agreement for each causal explanation were (in order from highest to lowest difference in agreement): *event* (0.12), *outcome* (0.07), *stimulus attribute* (0.06), *action* (0.06), *goal* (0.03), *disposition* (0.03), and *temporary state* (0.03). See **Figure 8** for an overview of the level of agreement in participants’ plausibility judgments of behavior causes.

## Conclusion and Discussion

The results show little variation in participants’ overall judgment of human vs. humanoid behavior (i.e., participants’ intentional stance toward the humanoid robot was in this case very similar to their stance toward the human actor). Firstly, we saw that the behavior enacted by the two types of agents were rated as similarly *intentional* and *desirable*. There was a statistically significant difference between ratings across conditions for *controllability*, with human behaviors rated as being more “under the actor’s control.” However, the effect size was relatively small at *d* = 0.26. Secondly, participants judged the seven mental causes as equally plausible explanations for the behaviors of the two agents in 46 out of 58 cases (80%). Thirdly, although humanoid behaviors were consistently rated with lower agreement than human behaviors, the level of agreement was very high in both conditions over most of the behaviors.

The high agreement in participants’ ratings of the humanoid robot points toward the existence of shared folk psychological conceptions of humanoid behavior. Moreover, the substantial overlap in ratings of human and humanoid behaviors indicates that people’s folk concept of human vs. humanoid intentional behavior may be composed of similar components – e.g., desire, belief, intention, awareness, and skill (cf. Malle and Knobe, 1997) – such that people view behavior as intentional only when they recognize these components in the behavior of the agent. The systematic differences found in participants’ ratings of the two agents point toward distinct differences in how people interpret robots and humans *qua* intentional systems. We now go into more detail discussing the results in relation to the specific research questions we have focused on in this paper.

### Q1: How **S**imilar or **D**ifferent **A**re **P**eople’s **J**udgments of the **I**ntentionality, **C**ontrollability, and **D**esirability of **H**uman vs. **H**umanoid **B**ehavior?

The results generally point toward a substantial overlap in participants’ judgments of humanoid and human behavior. Notably, there was no overall difference between ratings of *intentionality* and *desirability*, and there was a statistically significant but small (*d* = 0.26) effect on ratings of *controllability*. However, there were also some notable differential effects. Firstly, we saw that positive behavior was seen as more intentional when exhibited by the human than the robot. Negative behavior, on the other hand, received higher intentionality ratings when exhibited by the robot in three out of four cases. This finding seems to indicate that people see humanoid actors as more inclined to take undesirable (intentional) action (i.e., as more malevolent) and less inclined to act desirably (i.e., as less prosocial) than humans. The cause(s) of this inverse relation between the valence of behavior (positive or negative) and level of intentionality ascribed to humanoid and humanoid actors, respectively, is a topic that we believe warrants investigation in future work.

Secondly, as mentioned above, the effect of agent type on ratings of controllability (i.e., “the extent to which the actor’s behavior is under the actor’s control”) was relatively weak overall. However, the effects on two individual behaviors stood out as relatively strong compared to other behaviors: the positive state “Ellis is happy to be in the kitchen” and the negative action “Ellis lies about having cooked the dinner” (*d* = 0.76 and 0.84, respectively). That is, participants attributed considerably less control over lying and being happy to the robot. While we cannot presently offer an explanation for this finding we would like to note that these effects do not seem to have been influenced by participants’ ratings of the desirability of these behaviors. We would also like to point out that participants’ ratings of controllability did not seem to have been based on assumptions regarding physical restrictions in the body of the robot. For instance, one might expect that the relatively clunky fingers on the robot may have lead participants to infer less control over accidentally dropping a glass to the floor. As a suggestion for future work, the effect of morphological aspects of robots’ design on people’s judgments of the controllability robot behaviors could be controlled for in an experimental setup with a number of (morphologically) different types of robots.

### Q2: How Similar or Different Are People’s Judgments of the Causes of the Behavior of Humans vs. Humanoids, in Terms of the Underlying Folk-Psychological Mechanisms, and How These Overlap or Vary for Different Behavior Types?

The seven causes were judged as equally plausible explanations for the behaviors of the two agents in 46 out of 58 cases (80%). This is a strong indication that participants’ ascriptions to the robot were similar to that of the human case. The 12 observed differential effects were all moderately sized (ranging from *d* = 0.48 to *d* = 0.68) and the majority of them were related to judgments of *goal* and *disposition*. Goal was rated as a more plausible cause of behavior when the actor was human in four out of eight cases. In all of these cases goal was seen as a more plausible explanation for positive behavior (1 case) when exhibited by the human and more plausible for negative behavior (3 cases) when enacted by a robot. This resonates with the finding that participants saw positive behaviors as more intentional when exhibited by the human and negative behaviors as more intentional when exhibited by the robot. These corroborating results are not surprising given that “goal” is a constituent part of the folk concept of intentionality, i.e., people judge behavior as intentional only when they see it as goal-directed (Malle and Knobe, 1997). Hence, this points toward a distinct similarity in how people treat robots and humans *qua* intentional systems.

Disposition was rated (in five out of eight cases) as a more plausible cause of behavior when the actor was human. This raises the question whether people think of robots as less likely to have dispositions in the human sense, or as having less stable dispositions as humans, or whether people see robot dispositions as less efficacious in causing behavior than human dispositions. It should be noted, however, that we chose to translate “disposition” using the Swedish word “personlighetsegenskap” in order to approximate the meaning of the German word “Persönlichkeit” which was used in the psychological model on which we based our selection of explanation categories, i.e., the CEN model (Böhm and Pfister, 2015). “Personlighetsegenskap” can also mean “personality trait,” and differences between conditions might therefore have been influenced by a reluctance to ascribe personality to a robot.

### Q3: How Much Do People Agree or Disagree in Their Judgments of Humanoid Robot Behavior Compared to Judgments of Human Behavior?

The agreement in participants’ ratings was “excellent” for both agents with respect to ascriptions of intentionality, controllability, and desirability, as well as plausibility judgments of causes of behavior (with the exception of one case, *outcome*, which was rated statistically insignificantly differently by participants in the two conditions). This indicates that participants were in general *highly confident* in their interpretations of the robot as an intentional system. We also saw that participants consistently rated humanoid behavior with lower agreement than human behavior, which suggests that participants were *not as confident* in their intentional stance toward the robot as they were in their stance toward the human actor.

### Method

It is, of course, possible that subjects, while generally – in our experiments – attributing just as much intentionality to humanoid robot behavior as to human behavior, are reluctant to make certain attributions (e.g., an interest in “fantastic cake”). This is, very roughly speaking, the distinction between the previously discussed ascriptions of Dennett-type intentions (in the earlier illustrative example: Donald Duck is angry with Chip and Dale and wants to keep his pancakes) and the possession of actual Searle-type intentionality (Donald actually exists, has agency and genuine mental states). Keeping these two types of intentional attributions apart is an obvious challenge for experimental methodologies in the study of human social interactions with robots and other types of autonomous technologies.

The fact that questions given to participants in questionnaires are always (to some extent) open to multiple interpretation is an issue inherent in all questionnaire methodology. However, this might be particularly true in our case due to the technical character of terms such as “intention” and “disposition” which we chose not to supplement with any working definitions. The reason for not providing definitions for the terms used in the questionnaire was twofold: (1) we wanted to avoid biasing participant ratings and (2) previous work in attribution theory demonstrated that agreement in people’s ratings of intentional behavior remained unaffected when a working definition of intentionality was provided to them (Malle and Knobe, 1997). Nevertheless, we think that this issue warrants some caution when interpreting the results and therefore do not draw any strong conclusions based on ratings of individual questionnaire items. Another potential confound is that participants may have reinterpreted (semantically) the questions as a function of human vs. humanoid behavior stimuli, i.e., terms such as “intention” and “disposition” may have been assigned different meanings in the context of observing robot behavior. Indeed, it is difficult to disentangle between-subjects effects caused by differing judgments of the object from those caused by differing interpretations of the question. It should be noted that some degree of “stability” of concepts used in questionnaire items is assumed in all questionnaire methodology which generalizes across multiple interpreters. Our position here is that it is reasonable, as a starting point, to assume the absence of systematically different interpretations of questions across experimental conditions, since we have no a priori basis for assuming that the concepts used are acquire different meaning when they are ascribed to humanoid behaviors. Hence, we believe that there is no apparent risk that the reported between-group differences were influenced by systematic variation in interpretations of the questions across the two conditions. However, the existence of the above confound cannot be conclusively ruled out on basis of the study design and methodology used here, and we therefore propose that this could be a topic for future research.

### Future Work

In our future research, we intend to further develop the at this point admittedly relatively simple methodology used in this initial study of human vs. humanoid behavior. Further studies will not be limited to single pictures and sentences as representations of particular behaviors, but the stimulus material will be extended to deal with sequences of pictures, movies, and ultimately live interactions – in both virtual reality and the real world. As alternative measures of explanation, we consider using free-response questions as well as rankings or selections out of a set of explanations (cf. McClure and Hilton, 1997). Among other things, we also intend to apply this to a broader range of both natural and artificial autonomous agents – such as robots, virtual agents, or automated cars. This would help to contribute to addressing the broader and more fundamental question of how people’s social interactions with different types of agents are effected by folk-psychological causal explanations of observed behavior, and to what degree the underlying mechanisms overlap or differ for different types of natural and artificial agents. Needless to say, different types of artificial agents have their specific methodological constraints and challenges, but with the present study we hope to have made a significant step in the right direction and to have contributed toward the ambitious goal of overcoming some of the many conceptual and methodological limitations that currently characterize the study of people’s (quasi-) social interactions with different types of autonomous agent technologies.

In a broader perspective, it might be worth noting that, despite the fact that humanoid robots currently receive an enormous amount of attention in HRI research, popular science media, and science fiction, the question of when and how people take the intentional stance toward autonomous technologies is at least equally important to human interaction with autonomous systems that are not human-like at all, in particular automated vehicles. Given that many companies now are very actively working on putting such systems onto the market in the not too distant future, we believe that the question how people – e.g., in the case of (partially) automated cars: vulnerable road users, like pedestrians and bicyclists – will be able to (quasi-) socially interact with such systems, has significant societal relevance, and should receive more attention than it currently does (Nilsson et al., 2015; Habibovic et al., 2016). Hence, we also believe that HRI as a research area could benefit significantly from (a) generally carrying out more *comparative* research on how social interaction varies for different types of agents, and (b) more specifically, making more contact with research addressing how people interact with automated vehicles or other types of artificial agents that are more different to humans than the average social robot.

## Author Contributions

The work reported here is part of ST’s Ph.D. research under the supervision of TZ and AS. Accordingly, ST carried out the experiments and has written most of the text. All authors listed have made a substantial, direct and intellectual contribution to the work, and approved it for publication.

## Conflict of Interest Statement

The authors declare that the research was conducted in the absence of any commercial or financial relationships that could be construed as a potential conflict of interest.

## Footnotes

1 The Swedish terms used for the queries about behavior properties in questions 1–3 in the form were: *intentional*, avsiktligt; *under Ellis’ control*, under Ellis kontroll; *desirable*, önskvärt. The terms used as the Swedish translation of the explanation categories in Böhm and Pfister (2015), questions 4–10 in the form, were: *conscious goal*, medvetet mål; *action*, handling; *outcome*, resultatet av en handling; *uncontrollable event*, onkontrollerbar händelse; *temporary state (psychological or physical)*, tillfälligt tillstånd (antingen psykologiskt eller fysiskt); *disposition*, personlighetsegenskap, *attribute of someone or something in Ellis’ environment*, egenskap hos någon eller någonting i Ellis omgivning.

2 The two-way ICCs measuring “absolute consensus” reported here are technically a compound measure of both interrater agreement and interrater reliability (LeBreton and Senter, 2008).

## APPENDIX

**Table A1:** Correlations between aggregated ratings of each questionnaire item across all eight behaviors in human and humanoid (gray-marked) conditions.
